# Application of MEMS Accelerometers in Dynamic Vibration Monitoring of a Vehicle

**DOI:** 10.3390/mi14050923

**Published:** 2023-04-24

**Authors:** Hasnet Eftakher Ahmed, Sahereh Sahandabadi, Bhawya, Mohammed Jalal Ahamed

**Affiliations:** MicroNano Mechatronics Laboratory, Mechanical, Automotive, and Materials Engineering Department, University of Windsor, Windsor, ON N9B 3P4, Canada

**Keywords:** MEMS accelerometer, MEMS IMUs, data processors, automotive applications, road test, vehicle dynamics, vibration sensors

## Abstract

In this paper, the viability of MEMS accelerometers is investigated to measure vibration parameters related to different locations of a vehicle with respect to the automotive dynamic functions. The data is collected to compare the accelerometer performances in different locations on the vehicle, including on the hood above the engine, on the hood above the radiator fan, over the exhaust pipe, and on the dashboard. The power spectral density (PSD), together with the time and frequency domain results, confirm the strength and frequencies of the sources of vehicle dynamics. The frequencies obtained from the vibrations of the hood above the engine and radiator fan are approximately 44.18 Hz and 38 Hz, respectively. In terms of the vibration amplitude, the measured amplitudes are between 0.5 g and 2.5 g in both cases. Furthermore, the time domain data collected on the dashboard during driving mode reflects the road condition. Overall, the knowledge obtained from the various tests conducted in this paper can be advantageous for further control and development of vehicle diagnostics, safety, and comfort.

## 1. Introduction

MEMS (Microelectromechanical Systems) accelerometers are widely used in many fields, such as aerospace, automotive, industrial, biomedical, and consumer products [1], where they are required to sense acceleration. MEMS accelerometers are established components used in high volumes due to their unique features: low cost, wafer-scale fabrication, small size, and low power consumption. As a vibration sensor or structural health monitoring tool, a MEMS accelerometer collects acceleration data from regular or disturbed dynamic energy sources for reliability evaluation, operation, and fault diagnosis. For example, to detect the induced vibration from machine tools in the manufacturing industry, low-cost integrated MEMS accelerometers have been used [2]. Experiments have been performed to measure DC motor-induced vibrations from three commercial MEMS accelerometers [3]. Time and frequency domain data, along with wavelet transform, coherence, and power spectral density (PSD), were used for comparison with conventional piezoelectric accelerometers [3]. The performance of three different MEMS accelerometer was investigated in industrial CNC (Computer Numerical Control) machines, and the results were obtained from sinusoidal, impulse, and random excitation forces showed good agreement with the conventional piezo-type accelerometer results [4]. In another experiment, wireless MEMS accelerometers were attached to a hollow rotor, and subsequently, the vibration of the rotating frame was synchronously sensed and measured in industrial applications [5,6]. A MEMS-based accelerometer with a wireless board was used as a sensor for measuring vibrations on a pedestrian deck-stiffened arch bridge, providing results very similar to those of experimental studies [7]. In another study, low-cost MEMS accelerometers with an Arduino module were utilized to address the noise density and synchronization problem and then used to remotely monitor the structural health of bridges [8]. Apart from vibration monitoring of machines discussed above, MEMS accelerometers are also used to monitor vibrations in automotives. 

**Figure 1:**
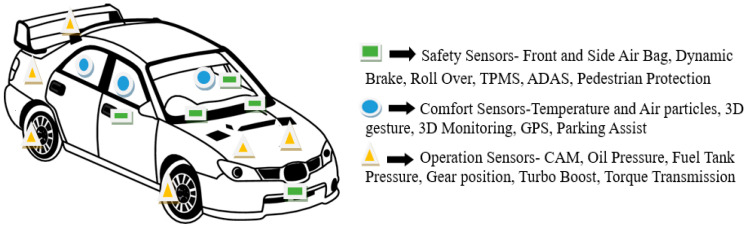
Deployment of MEMS sensors in potential smart vehicles.

Utilizing diverse types of sensors and actuators, including accelerometers, in the automobile industry is common, as vehicles need smooth controllability, high safety, balanced stability, and visual monitoring. For many years, piezoelectric sensors have been used for different purposes, such as improving engine performance, tire pressure sensing, vehicle safety, and emission control for environmental protection. The size, cost, performance, and accuracy of sensors must be considered constantly so that they are desired in the competitive automotive industry. MEMS sensors have emerged as the preferred solution for addressing various inertial sensing application because of their accuracy, reliability, smaller size, and cost-effectiveness [9,10]. MEMS sensors are being used in the auto industry as single-purpose or multipurpose sensors, including pressure, temperature, speed, position, and flow measurement sensors [11]. Among the mentioned sensors, the MEMS accelerometer is more prevalent in the auto industry for different purposes due to its promising functioning characteristics. For passenger comfort and seating dynamics research, a compact system of commercial MEMS accelerometers was used to store the experimental vibration time domain data for seat improvement and performance evaluation [12]. It is also used to study vehicle interior vibration, vehicle characteristics, speed, and traffic conditions [13]. Driving control systems of autonomous vehicles or self-driving cars are recent concepts of advanced car designs that include MEMS accelerometers [14]. For many years, a wide range of MEMS sensors and actuators have been utilized to achieve specific objectives, such as electronic stability control (ESC), rollover and skidding detection, engine component management, pressure monitoring, and security, among others [15]. In the 1990s, the application of g-switches in the airbags of vehicles became common to provide protection from the impact force of a sudden accident. However, there were concerns regarding the size, weight, and cost of g-switches. Shortly thereafter, MEMS accelerometers hit the market as alternatives to g-switches. MEMS accelerometers can sense and coordinate with electronic stability control (ESC) systems for a quick and accurate airbag response. They are very common in automotive airbag applications due to their easy manufacturability, accuracy, and low cost. In addition to their application in airbags, MEMS-based accelerometers have been used for crash detection and multiple airbag deployments for an impact force ranging from 6 g to 18 g [16]. A capacitive signal from the MEMS accelerometer’s output is sent to the ESC, which coordinates the airbag deployment during an accident. In addition, security and antitheft systems can use MEMS accelerometers to detect impacts, vehicle body tilt, and movement [17]. MEMS accelerometers can track the vehicle noise to check if the engine is running or idle, measure the impact intensity on the vehicle body, measure the acceleration or deceleration from braking, and measure the inclination of the vehicle body. The ESC and anti-lock braking systems use acceleration measurements to offer reliable safety in the driving experience. By filtering the MEMS accelerometer’s unprocessed data from multiple-axis acceleration measurements with random noise, the position of the vehicle and longitudinal velocity can be obtained [18]. Vehicle accident monitoring is possible by triggering pulses from the MEMS accelerometer at regular intervals to the controller utilizing the Global System for Mobile (GSM) communication, GPS (Global Positioning System) modem, and Subscriber Identity Module (SIM) mounted on vehicles. This process can help track the vehicle constantly in any adverse environment [19]. The application of GPS, GSM, and the MEMS accelerometer for air gravity lock recognition systems was developed for next generation smart vehicles that can detect and prevent auto theft [20]. MEMS accelerometers, together with GPS, can be used for a nonspecific user gesture recognition system, which can be used as an auto theft prevention system for smart car development [21]. To increase the safety and security of modern smart cars, MEMS accelerometers and ultrasonic sensors have been used for vehicle tracking, vehicle monitoring, accident alerts, and antitheft prevention [22]. The effectiveness of accelerometers and gyroscopes for unbalanced detection purposes has been investigated [23]. Measuring the pavement roughness with the help of vehicle-mounted MEMS accelerometers is efficiently possible because of their accuracy. An integrated and wireless transfer-based system with GPS was developed to collect data from a MEMS accelerometer, which can be used to obtain the pavement roughness index [24]. Pavement irregularity was measured by spectral data analysis of vehicle vibration response data [25]. The efficacy of an electronic data acquisition system comprising an Arduino board, a low-cost microcontroller, which effectively collected data from MEMS accelerometers used for measuring automobile dynamics, was demonstrated in [26]. A mechanism was developed in [27] to obtain the concurrent kinematic state of a vehicle by using the accelerometer data from a smartphone mounted on the vehicle. A low-cost data acquisition system, including a Raspberry Pi and MPU6050 IMU accelerometer was used to collect data from soft/cruising and hard/accelerating, hydraulic-brake, and engine-brake behaviors [28] Vehicle powertrain mounting system vibration and noise were improved by performing a multi-excitation test and analyzing stiffness optimization [25,29]. A self-balancing platform for smart vehicles was developed using MEMS accelerometers and other navigation parameters [30]. Although MEMS accelerometers and navigation data can be obtained and analyzed for various purposes, the data are not noise-free or free from temperature impact. An error compensation method for MEMS accelerometers was presented for vehicle navigation and testing system data collection [31]. The trustworthy Advanced Driver-Assistance Systems (ADAS) and Automated Vehicles (AV) systems were linked to the accuracy and reliability of MEMS sensors, including MEMS accelerometers. A digital 3D gyroscope integrated into a commercial multisensory MEMS system was developed and tested with dynamic reference acceleration [32]. To compensate for the high-temperature frequency stability of silicon, MEMS doping can be used to make the MEMS sensor more temperature stable and suitable for harsh environments in automotives [33,34]. Numerous other applications of MEMS accelerometers have been presented for automotive applications [35]. However, MEMS accelerometers can also be used to capture various other dynamic vibration conditions, including seat, steering wheel, dashboard, radiator, exhaust, etc., and for operation, diagnostics, comfort, control, and safety as shown in Figure 1.

An adequate amount of measurement data is needed from different spots on the vehicle to provide information about safety, comfort, and reliability. Concisely, many researchers have studied, designed, tested, and analyzed data obtained from different laboratory and commercial MEMS accelerometers. Despite the importance of improving and developing smart vehicles equipped with various accelerometers, a comparative study between different MEMS accelerometers has not been conducted to analyze dynamic test results in automotive applications. Such a study could provide valuable insights and a range of dynamic data. In this study, the dynamic test results of an in-house MEMS accelerometer and three commercial IMU accelerometers are presented. The in-house accelerometer sensor is a MEMS capacitive, single-mass, classical resonating accelerometer device. The device is fabricated by a commercial double SOI (silicon on insulator) wafer-based fabrication process known as the MicraGEM-Si process. The details of the SOI-based fabrication process are available in [36,37]. Its measurement range is ±8 g with an excitation voltage of 5 V. The in-house sensor output, which demonstrates the signal sensed from the vibrations, is obtained from the vehicle using lab-built circuitry. In Section 2, information about the commercial IMU MEMS accelerometers and microcontrollers used in this paper is provided. Moreover, the experimental setup, test plan, and data acquisition process are briefly described in Section 3. Section 4 includes the results of the data analysis in detail. Next, in Section 5, the MEMS accelerometer and its role in modern auto industry applications are discussed. Finally, the conclusion is presented to summarize the most interesting findings of this paper.

## 2. Sensors and Test Setup

**Figure 2:**
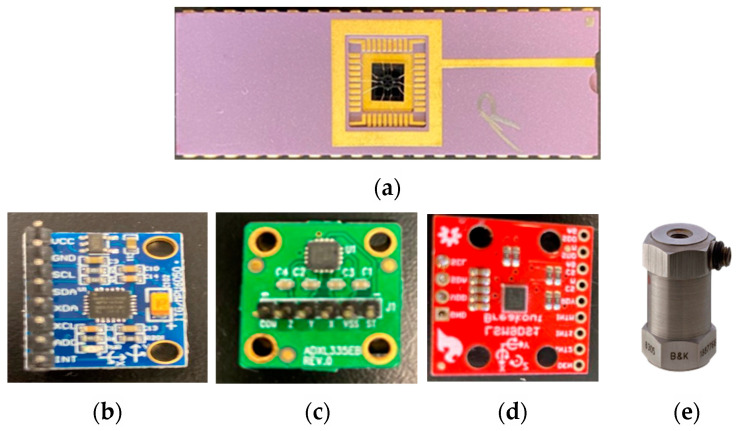
(**a**) In-house MEMS accelerometer chip and commercial IMUs with accelerometers (**b**) MPU6050, (**c**) ADXL335, (**d**) LSM9DS1, and (**e**) Piezoelectric 8305.

The MEMS accelerometers used in the current experiment and their basic scheme, together with standard reference accelerometer specifications, are displayed in Figure 2 and Table 1.

The sensors are compared to the reference standard 8305 piezoelectric accelerometer. The measurement and temperature range are higher in 8305. These advantages come with the downside of non-customizability. Choosing silicon in MEMS accelerometers over quartz in piezoelectric sensors provides survival against shock and vibration. Correspondingly, the higher failure rate of quartz versus silicon can add to the costs for root-cause analysis, repairs, and replacements.

**Figure 3:**
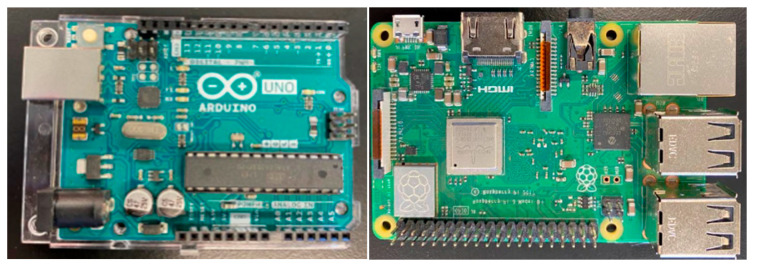
Commercial data processors Arduino UNO (**left**) and Raspberry Pi (**right**).

The commercial microcontrollers used in this experiment are displayed in Figure 3 and are used for data collection from the sensors. Their technical data are provided in Table 2 and Table 3.

**Figure 4:**
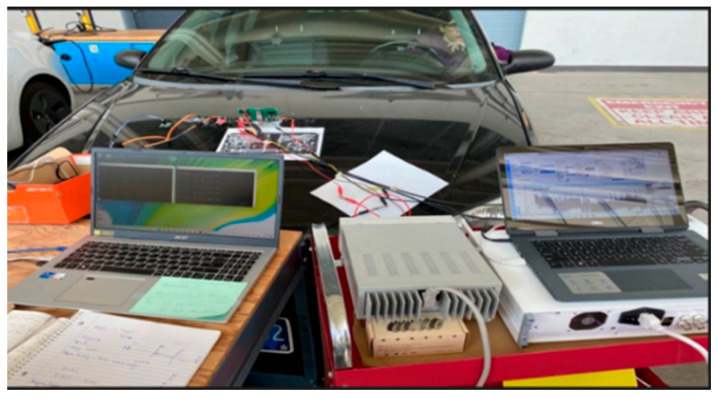
The experimental test setup to measure the dynamic data of the sedan vehicle.

The MEMS accelerometer data is collected while the sensor is mounted on the vehicle. The experimental setup is conducted at the University of Windsor Automotive Research Lab and Micro-Nano Mechatronic Lab shown in Figure 4. For driving mode data collection, vehicles are tested on city roads to acquire reliable and realistic dynamic data for real-world environments. In the lab experiment, the performance of the laboratory sensor was assessed by exciting the driving terminal and measuring the output from the sensing terminal using a sense and drive circuit. This process was similar to the one presented in [38].

The LSM9DS1 sensor (STMicroelectronics, Geneva, Switzerland) is connected to the Raspberry Pi B plus. The data is collected using the Serial Data (SDA) and Serial Clock (SCL) pins of the Raspberry Pi. MPU6050 (TDK InvenSense, San Jose, CA, USA) is connected to the Arduino Uno board (Monza, Italy), and through its SDA and SCL channels, the data is collected. The sensitivities of the analog and digital accelerometers are expressed in mV/g and LSB/g (least significant bit per unit of acceleration), respectively. The MPU-6050 has an operating voltage of 1.8 V with an analog-to-digital conversion (ADC) scale of 16 bits. Thus, each unit of LSB is equivalent to 0.0275 mV, and 16,384 LSB/g is equivalent to 450 mV/g [39]. Using the mentioned conversion scheme, the MPU data have been converted to mV/g so that the comparison of results to other IMU accelerometer results provides a meaningful explanation. The ADXL335 (ElectronicWings, Pune, India), an analog sensor not reverse polarity protected, is connected to the analog (A0, A1, A2) pins on the board.

Various spots on the vehicle are considered for mounting sensors. These spots have been deliberately selected. The study of hood vibrations on the engine or radiator fan can provide valuable information on the improvement of hood performance in accidents as well as health monitoring of the engine and the radiator fan. The dashboard vibrations are directly related to the passenger comfort level and vibration isolation from sources outside the passenger/driver area. The exhaust pipe vibration can be an indication of an exhaust leak. This paper will discuss multiple reasons for testing the sensors at the abovementioned locations. The unprocessed data from the in-house MEMS accelerometer are at the signal level (mV), and commercial IMU MEMS accelerometer data are expressed in acceleration (g). As discussed before, the conversion of the units is crucial to enable us to compare the sensitivity in both cases. Since this paper focuses on employing MEMS accelerometers in different environments, the experiment is conducted in both idle and driving modes.

(1) The vehicle is idle, and accelerometers are mounted in different positions on the vehicle, including:

(a) on the hood above the engine, (b) on the hood above the radiator fan, and (c) on the trunk and the exhaust pipe.

(2) The vehicle is in driving mode, and accelerometers are mounted on the dashboard in three road conditions:

(a) local roads with frequent braking, (b) highways with high speed, and (c) a bumpy country road.

Both in-house and commercial MEMS sensors sense in-plane vibration. The data is collected for a 120 s duration, from the hood above the engine, the hood above the radiator fan, over the exhaust pipe, and on the dashboard of the tested sedan car. The 120 s are split into 3 sections: engine off before turning on the car (40 s), idle while the vehicle is on (40 s), and after turning the vehicle off (40 s). A detailed test plan is presented in Table 4.

## 3. Data Acquisition, Results, and Analysis

Experiments are conducted using a sedan car, and dynamic data have been collected using an in-house MEMS accelerometer and IMU MEMS sensors for both idle and driving modes. The in-house sensor is mounted on the car using a customized in-house-designed test fixture. The commercial sensors are mounted using duct tape. The mounting is checked to ensure proper orientation for all sensors. Data from the lab experiment was collected under controlled conditions, and data were not collected from the in-house sensor on the road test due to the difficulties related to using the instruments to collect the data. The laboratory temperature was 23 °C during the experiment, which is within the operation temperature range of commercial accelerometers. The temperature on the exhaust pipes and radiator fan was also within the normal operation range because the vehicle was only on for two minutes, and the next test was minutes later. Therefore, there was enough time for the exhaust pipe and the hood to cool down and return to room temperature before the next test started. For the abovementioned reasons, the temperature parameter is not considered in the data analysis. The noise effect is within the normal range of the everyday use of a vehicle. The test is run before starting the car for 20 s to check the noise level. As seen from the results, the noise in the normal test environment can be neglected because of its much lower level than the signal level.

### 3.1. Dynamic Data of the Vehicle in Idle Engine Mode

Dynamic data of various positions on the vehicle are collected in idle engine mode. The time and frequency domain results of the experiments are presented in the subsequent sections.

#### 3.1.1. Accelerometers on the Hood above the Engine

**Figure 5:**
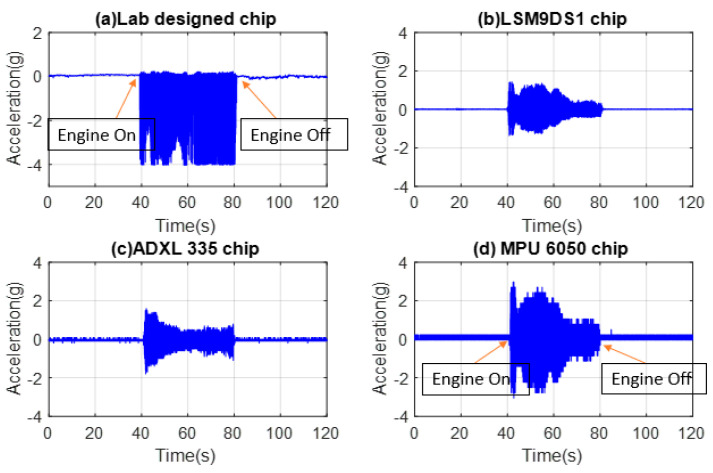
Time-domain data of various MEMS accelerometer sensors used in this study: (**a**) in-house, (**b**) LSM9DS1, (**c**) ADXL335, and (**d**) MPU6050.

The time-domain data were collected by mounting the chips on the hood above the engine, plotted, and presented in Figure 5a–d and d for the lab designed in-house sensor, LSM9DS1, ADXL335, and MPU6050, respectively. The comparison of laboratory and commercial IMU MEMS accelerometer data plots indicates a similar trend with few discrepancies, which might have occurred due to the different sensitivities of the related sensor. The in-house and MPU6050 sense higher values of acceleration compared to ADXL335 and LSM9DS1. The signal level in mV can be converted to g units using sensitivity data.

**Figure 6:**
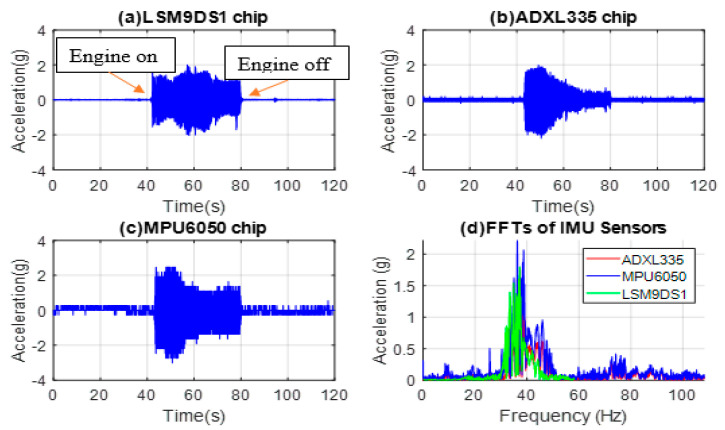
MEMS accelerometer chip data from the hood above the radiator fan for (**a**) LSM9DS1, (**b**) ADXL335, and (**c**) MPU6050, (**d**) FFTs of all three sensors’ signals.

The maximum deviation of sensitivity is described by the frequency response. The frequency response provides the deviation over a frequency range. The sensitivity itself, however, is measured at a particular frequency, which is generally lower than the mechanical resonance of the sensor. Therefore, the different measured acceleration amplitudes seen in Figure 5 for the same experimental condition are due to different frequency responses of the sensors. As seen in Figure 6d, the accelerometers sense different ranges of frequencies. Moreover, there are other factors that can be behind different measured accelerations, such as noise, linearity, and temperature tolerance.

Very low amplitude vibrations (mostly noise signals) can be observed for 40 s prior to starting the car. The vibration amplitude increases after starting the car, and the g values subsequently increase. The LSM9DS1 and ADXL335 sensor time data are presented, indicating a gradual decrease as the engine vibration settles down. After turning the engine off, the signal levels abruptly decrease, taking 40 s and expressing lower-level vibrations. The sensors have reliable data for sensing the vibrations when the engine starts and stops. Among commercial IMUs, the MPU6050 response has a higher amplitude compared to the ADXL335 and LSM9DS1 IMUs.

#### 3.1.2. Accelerometers on the Hood above the Radiator Fan

Similar to the previous tests, IMUs are mounted on the hood above the radiator fan, and the time domain data were collected and converted to acceleration (g) presented in Figure 6a–d for LSM9DS1, ADXL335, MPU6050, and the FFTs of the three commercial sensors, respectively.

The signal output is in g in this case since all the sensors are commercial ones with signal levels ranging from 2.2 g to 2.5 g in idle engine mode. The plots suggest that the ADXL335 and MPU6050 sensors respond similarly as the vibrations gradually decrease. From the FFT and PSD plots, resonance frequencies of 38 Hz and 75 Hz are obtained for the engine and radiator fan tests, respectively. As seen in Figure 6, the signal level obtained from the MPU6050 sensor is higher. A total of 2 different frequencies are sensed here, 38 Hz being the dominant frequency, which corresponds to 2280 RPM from the radiator fan. The higher frequencies are more indicative of the structural health of the subsystems in the engine. It is noteworthy that in this type of vibration test, the results depend on what one is looking for and help understand the sources of the vibrations.

#### 3.1.3. Accelerometers on the Exhaust Pipe

**Figure 7:**
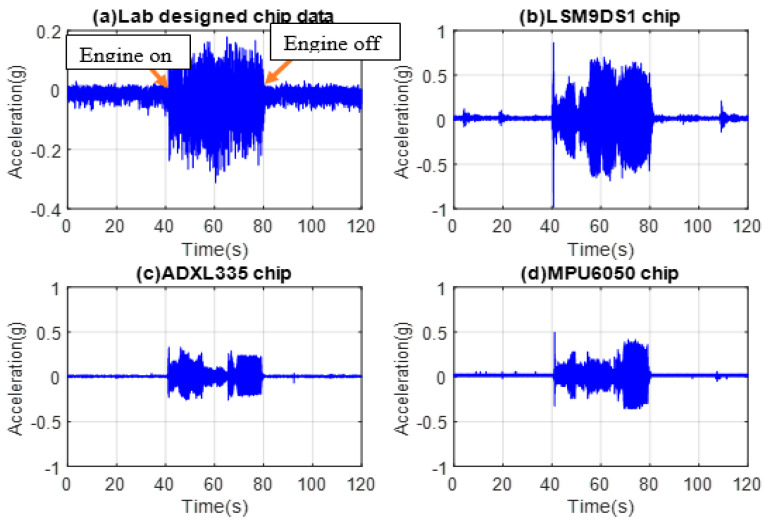
Time data of the (**a**) in-house, (**b**) LSM9DS1, (**c**) ADXL335, and (**d**) MPU6050 accelerometers from the exhaust pipe.

IMU accelerometers are mounted on the exhaust pipe. The measured time-domain signals are presented in Figure 7a–d for the laboratory, LSM9DS1, ADXL335, and MPU6050 sensors, respectively. The signal level obtained from the exhaust pipe test is in mV and was measured using a lab-designed sensor. The amplitude of the signal ranged from −100 mV to 50 mV and was then converted to g. The data collected for commercial sensors were in the range of 0.6 mV to 0.8 mV while the engine was on. The noise of the lab-designed plot is high due to the circuitry and sensor sensitivity. The test results suggest that, compared to the in-house chip, the commercial sensors respond more sharply to increases and decreases in vibration, as indicated by the sharper changes in the signal level.

#### 3.1.4. Accelerometers on the Dashboard in Idle Mode

**Figure 8:**
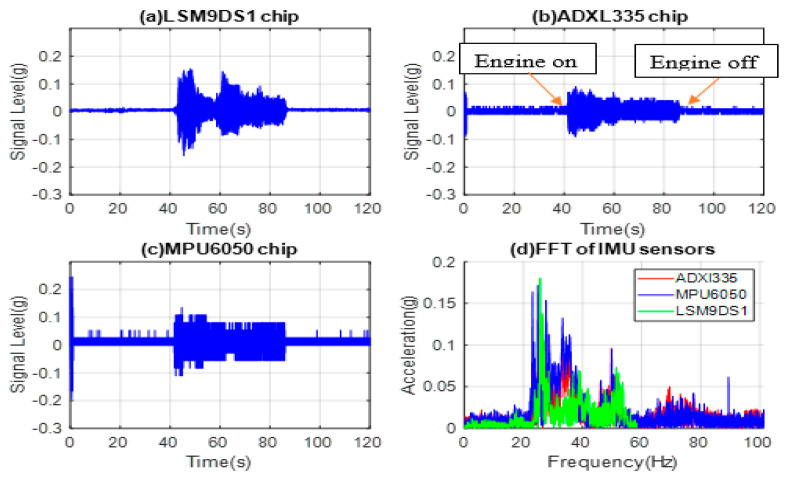
Tested data from dashboard-mounted commercial accelerometers (**a**) LSM9DS1, (**b**) ADXL335, (**c**) MPU6050, and (**d**) FFTs off all three sensors’ signals.

The dashboard data are directly related to the passenger comfort level. IMU accelerometers are mounted on the dashboard in the front seat and passenger side. The acquired time-domain data was then converted to the acceleration in (g) and presented in Figure 8a–d for LSM9DS1, ADXL335, MPU6050, and FFTs of all three commercial sensors, respectively.

The data is collected on the dashboard in idle engine mode. There are lower-level vibrations before starting the car. By starting the car, the vibrations grow higher in amplitude. Therefore, there is a sharp increase in the acceleration value at 24 Hz frequency. The time-domain data show a gradual decrease in engine vibration as the engine settles. By stopping the engine, the signal level decreases abruptly, and for that reason, in the last 40 s, there are only very low-level vibrations. The 24 Hz frequency of the vibration is related to the vibration sensed inside the car by the passenger, which is the superposition of all vibrations after being carried and attenuated from the engine to the dashboard. The intensity of the vibrations is also attenuated because of some level of isolation from the engine.

#### 3.1.5. MEMS Accelerometer Data of All Positions

**Figure 9:**
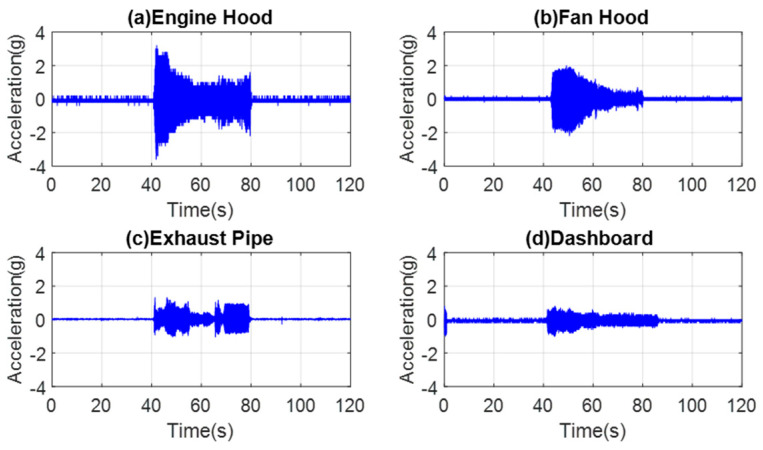
ADXL335 accelerometer mounted at different positions: (**a**) engine hood, (**b**) fan hood, (**c**) exhaust pipe, and (**d**) dashboard.

Finally, ADXL335 data collected from the hood above the engine, hood above the radiator fan, exhaust pipe, and dashboard positions are compared to provide a better understanding of the strength of dynamic sources presented in Figure 9a–d. The vibrations are high in amplitude at the time of engine ignition; hence, a sharp increase in acceleration values is seen before gradually decreasing as the engine settles. Figure 9 shows that the amplitude of vibrations on the hood above the engine is higher than the amplitude of vibrations on the hood above the radiator fan and exhaust pipe. The results show the lower amplitude of the vibrations on the dashboard since they are attenuated when carried from the engine to the dashboard.

As seen from Figure 9, the pattern of settling down is similar in three tests on the hood above the engine, hood above the radiator fan, and dashboard because they are directly proportional to the vibrations from the engine and the fan as a whole system. However, the exhaust pipe has an independent pattern because it is far from the engine and its vibrations.

#### 3.1.6. In-House MEMS Accelerometer on the Hood above the Engine

**Figure 10:**
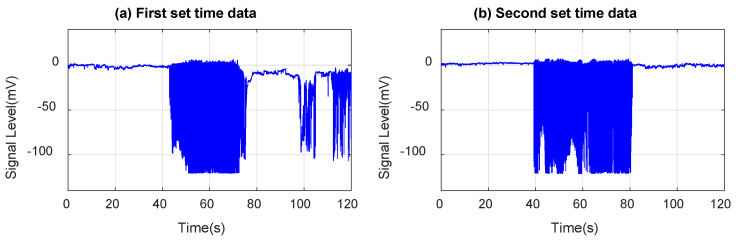
Time domain response test data (from the engine) of lab-designed in-house MEMS accelerometer chip: (**a**) first set of time data; (**b**) second set of time data.

The in-house MEMS accelerometer data is collected by mounting the chip on the hood above the engine. Two sets of data are collected and presented in Figure 10. When the engine is off, only noise is detected. At 40 s after starting the car, the chip senses vibrations caused by the engine, and this continues until the engine is turned off at 80 s. After the engine is turned off, the chip no longer detects any vibrations except for noise. In some tests, the noise is stronger than in the other tests because of the noise sources that are not controllable, such as the passing cars around the lab building. The plots demonstrate that the sensors are adequately sensitive and capable of producing a response. As previously explained, the output signal from the in-house sensor is in mV due to its analog nature and has also been normalized.

**Figure 11:**
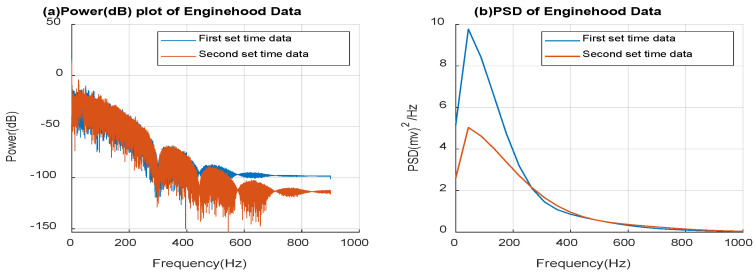
(**a**) Power (dB) and (**b**) PSD of measured data from vibrations on the engine by the in-house sensor.

Moreover, the acceleration signal output is in mV, and the peak-to-peak range is approximately 122 mV. Figure 11 indicates that the sensor responds consistently. Since the amplitude of the response is in the mV range, the signal strength is sufficient to account for small ranges of movements. Power and PSD profiles are derived from the engine hood time response data to reflect the signal power and energy density versus frequency, as shown in Figure 11.

The PSD helps to understand the strength of the signal. Because of multiple frequencies present in the sensor signal, which itself comes from vibrations, there are various peaks in the PSD plot. The relationship between the signal and power in dB is given by power (dB) = 10log_10_(y), where y is the PSD of the signal. The signal strength decreases gradually over the frequency range, which implies that most of the vibrations from the engine are of a low-frequency nature. From Figure 11b, the peak amplitude is at the 44.18 Hz frequency for the presented two sets of data. The vehicle on which the tests are run has an I4 engine (4-cylinder engine) and an RPM of 2500. Since only the dominant vibrations are of interest in this test, a sampling frequency of 200 samples/sec is used. The vehicle under test (VUT) has 2500 revolutions per minute (RPM), which corresponds to 41.67 Hz since 1 RPM represents 1/60 Hz. The analytical 41.67 Hz is accurately sensed by the in-house sensor at 44.18 Hz. Most of the signal power is present below 200 Hz. Nevertheless, there are some broadband vibrations over 200, which is an indication of subsystems. This is particularly significant when studying structural health, as it can provide valuable information about potential failures and aid in the diagnosis of such failures. Nonetheless, in this paper, we are most interested in the lower frequency vibrations related to the comfort level and the validity of the vibration tests.

### 3.2. Dynamic Data from the Dashboard of the Vehicle in Driving Mode

While the vehicle is in drive mode, the data is collected for 120 s by mounting all accelerometers on the dashboard of the sedan car. There is a combination of different vibration sources affecting the dynamic data collected from mounting the sensors on the dashboard. This experiment is divided into three sections. First, data is collected while the car is being driven on local roads with local posted speed limits and several stop signs that need the vehicle to come to full stops. Then, data is collected from highway driving, and data recording starts just before the ramp or speeding to adjust to the high speed and stops by exiting the highway. Finally, the data is collected from country roads where the roads have many potholes and bumpy areas.

#### 3.2.1. Dynamic Data Measured on Local Roads

**Figure 12:**
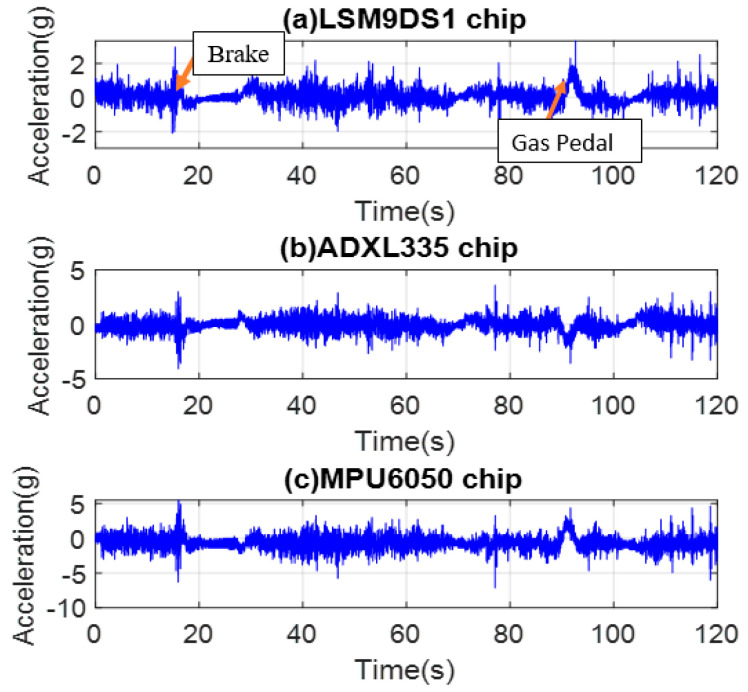
MEMS accelerometer data in the local road (**a**) LSM9DS1, (**b**) ADXL335, and (**c**) MPU6050.

Commercial IMU accelerometers are mounted on the dashboard. Then, time domain data are collected with acceleration in g and presented in Figure 12a–c for LSM9DS1, ADXL335, and MPU6050, respectively. Figure 12 depicts the time-domain data collected from the vehicle while driving on local or residential streets with a speed limit of 50 km/h and high traffic congestion, using sensors mounted on the dashboard. The impact of unevenness on these roads, particularly the need for full stops at stop signs, can be seen from the plots. It can be concluded that all three IMUs with MEMS accelerometers exhibit consistent sensitivity and data storage.

Whenever brakes are pressed, the vibrations increase sharply and then settle down for a while during rest time, and again, when the gas pedal is pressed, the vibrations increase. This can be seen from the plots in Figure 12, where the signal is recorded and displayed. The vibration condition of the vehicle depends on the road roughness, suspension type of the vehicle, road traffic, and driving conditions. Vibration amplitudes higher than 2 g are deemed uncomfortable, occurring a couple of times in the road test, which is expected because of the car type and model used in the test and a couple of potholes during the ride.

**Figure 13:**
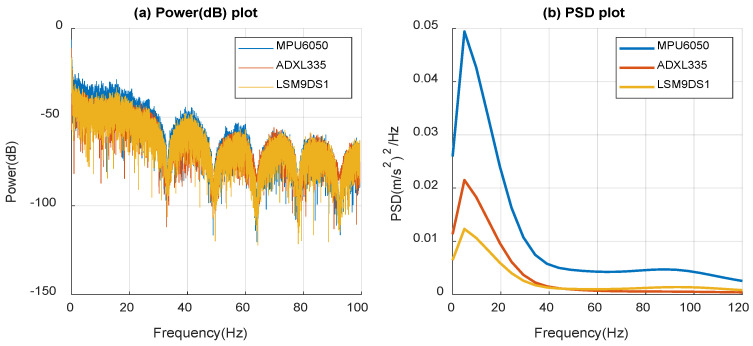
(**a**) Power (dB) and (**b**) power spectral density (PSD) of measured data from the local driving.

Figure 13 shows the power (dB) and PSD profiles obtained from the dashboard time response data while driving on local roads. These profiles have been used to observe the signal power and energy density in various frequency spectra. Power and PSD values have been compared for three commercial datasets, where it is observed that MPU6050 has a better response compared to ADXL335 and LSM9DS1. This happens due to the range of the acceleration measurement of the sensors. The strength of the signal gradually decreases versus the frequency spectrum, which means that the low-frequency components are stronger. All three sensors have very similar behavior over the displayed frequency range. The peak response is observed at 5.03 Hz for all 3 sensors, as shown in Figure 13b. Under local road conditions, most of the signal power is present at frequencies below 20 Hz. In, Figure 13a, there were some less strong broadband vibrations over 20 Hz. It is important to note that the vibrations experienced during road tests are much stronger and at lower frequencies, resulting in less power being present in the higher frequency ranges and should not be confused with the current case.

#### 3.2.2. Dynamic Data from the Highway Test

**Figure 14:**
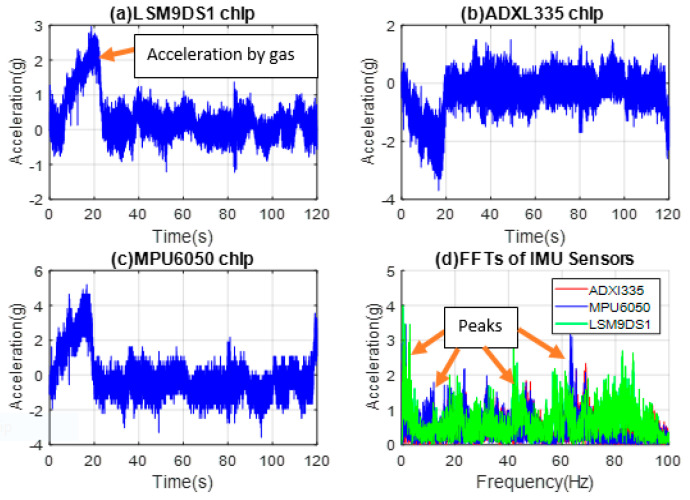
Accelerometer response in highway mounted on dashboard for (**a**) LSM9DS1, (**b**) ADXL335, (**c**) MPU6050, and (**d**) FFTs of all three sensors’ signal.

Commercial IMUs are mounted on the dashboard with the time domain data represented in acceleration (g) in the highway road condition seen in Figure 14a–d for LSM9DS1, ADXL335, MPU6050, and FFT plots of all three commercial sensors, respectively. Figure 14 shows the time data of the same vehicle on a highway with a speed limit of 100 km/h and high traffic flow while the sensors are mounted on the dashboard. The sharp increase in the acceleration can be seen in Figure 14 before entering the highway and then settling gradually once on the highway.

For the driving test on the highway in real-world conditions, the experiment is conducted to represent the performance of all three commercial sensors for a 120 s time duration. The car acceleration accounts for the first 20 s of data where the vibrations are the strongest. As the ride becomes smoother, the acceleration in g decreases. By reaching a speed of 100 km/h and maintaining the same speed for an adequate amount of time, the vibrations are all notably constant from 20 s to 110 s. The FFT analysis of the sensor data indicates that the low-frequency vibrations (approximately 5 Hz) detected by the sensors are predominantly from the normal road surface (such as highways and local roads with few potholes). The sensors capture the acceleration dynamics from pressing the gas pedal. In contrast, the FFT plot exhibits higher frequency and lower amplitude vibrations emanating from the vehicle itself. During the road test, the frequencies observed are below 20 Hz, while those above 20 Hz are attributed to engine and highway driving conditions. These findings underscore the importance of considering frequency spectra when analyzing vibration data from road tests, particularly when discerning between the road surface and vehicle-generated vibrations.

#### 3.2.3. Dynamic Data from Tests on Bumpy Roads

**Figure 15:**
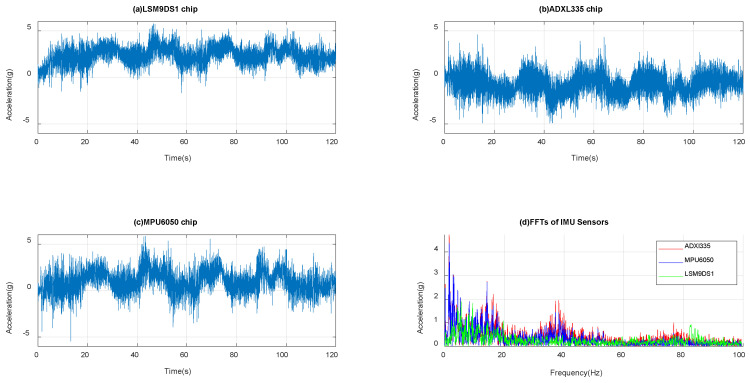
Accelerometer data in bumpy country roads for (**a**) LSM9DS1, (**b**) ADXL335, (**c**) MPU6050, and (**d**) FFTs of all three sensors’ signals.

The commercial IMU are mounted on the dashboard, and the time domain data are presented in acceleration (g) shown in Figure 15a–d for LSM9DS1, ADXL335, MPU6050, and all FFTs together, respectively. Figure 15 shows the unevenness of the driving track. All three IMUs with MEMS accelerometers have stable sensitivity and data storage. In this case, while driving on a gravel or uneven road, the vibrations are primarily of low frequency, as observed through FFT data analysis. The FFT data from the bumpy roads indicate multiple resonance peaks with high amplitude at low frequencies and low amplitude at high frequencies, indicating an appropriate response to both the road surface and vehicle dynamics. The time plot fluctuations correspond to vibrations caused by potholes of various sizes and their recurrence.

**Figure 16:**
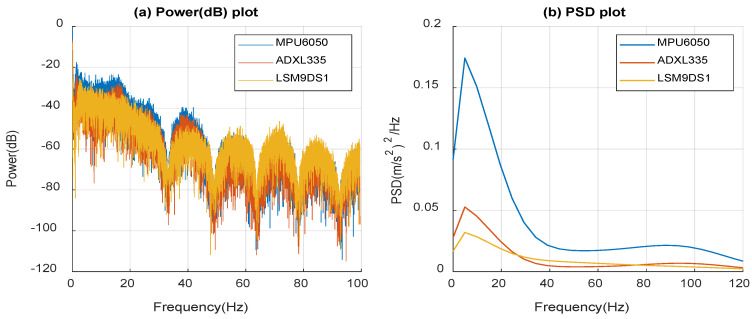
(**a**) Power (dB) and (**b**) power spectral density (PSD) of measured data from the bumpy road condition.

Power (dB) and PSD profiles are derived from the dashboard time response data while driving on the bumpy road to observe the signal power and energy density with different frequency spectra, as shown in Figure 16. By comparing the three commercial sensors’ data, MPU6050 has a better response compared to the other two sensors. This is due to the range of the g-measurement of the sensors. However, the response is stable for all three sensors over the frequency spectrum. The strength of the signal gradually decreases in the three sensor response plots, displaying similar behavior over the frequency range. More peaks are visible in Figure 16a because the signal has more vibrations. At 4.88 Hz, all 3 sensors in Figure 16b show a peak response, which means that the current vibrations have a very low frequency. This low-frequency nature of the vibrations is consistent with all road tests, which have also shown that vibrations in the road environment are of a low-frequency nature. The second strongest frequency occurs at approximately 18 Hz, which is overlayed by the bumpy road condition. In the bumpy road test, in contrast to the local road test, the amplitude of vibrations is higher, as shown in the PSD plot in Figure 16a. This is consistent with the fact that there are stronger motions on bumpy roads.

## 4. Significance of MEMS Accelerometers in Automotives

The main application of MEMS accelerometers in the automotive industry is airbag sensors for crash detection used by manufacturers because of their low cost and availability. Nevertheless, these sensors can also be used as vibration sensors to detect the dynamic conditions of automobiles. Vibrations from different locations in a vehicle can create a level of discomfort for the passenger. Quantification of the vibration data from inside the car can provide more information about the overall comfortability and reliability of vehicles. It is possible to determine the speed and condition of a vehicle from the acceleration fluctuations and angular rate changes while the vehicle is in off, idle, or driving mode. This can be completed using several types of MEMS devices, such as gyroscopes, speed sensors, and position sensors, including MEMS accelerometers. Some applications are described in the literature, which is seen in advanced car models, although some have recently become more prevalent than before. This paper investigates the dynamic conditions of a vehicle. The acceleration data are collected using a laboratory MEMS accelerometer and three commercial MEMS accelerometers. The data collected in idle mode can be considerably beneficial for future vehicle developments. Additionally, data obtained from road tests can be used to provide meaningful information about road conditions. The amplitude of time data shows the real scenario of the driving condition, and the same time data are used for FFT plots.

The dominant frequencies are provided for each location on the vehicle in Table 5. The most dominant frequencies are under 100 Hz, showing the low-frequency behavior of the vibrations for the tested vehicle. Analyzing the frequency and amplitude of vibrations helps determine which vibrations have a greater impact on the comfort of passengers in a car. While the strongest vibrations occur from the engine, it is important not to overlook the radiator fan vibrations. Despite being farther from the passenger seat, these vibrations have high amplitude and a slightly lower frequency that can affect the distance they travel. As a result, they can contribute just as much as the engine vibrations.

Various frequencies are present because of the variety of parts and subsystems in the vehicle. For each location, there is a main resonant frequency. However, in the hood above the fan and exhaust pipe cases, there is a second oscillating frequency accompanying the main frequency, which is the same for all sensors. The presence of the second oscillating frequency is due to the other system components near the fan and exhaust pipe.

**Figure 17:**
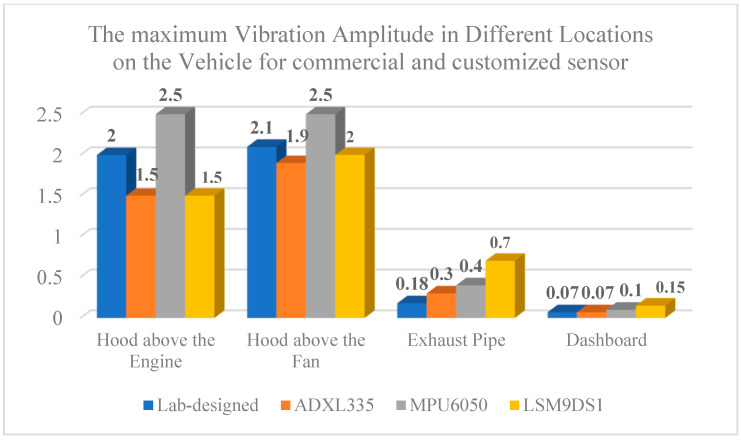
Comparison of the maximum vibration amplitude in different locations on the vehicle.

As seen in Figure 17, the strongest motions are present in the engine and radiator fan. There is a level of isolation from the engine to the dashboard, which leads to lower levels of vibrations.

In [36], the vibration data are collected from a 2006 Renault Clio 1.5 L Diesel car (Boulogne-Billancourt, France) on the dashboard using quickDAQ version 1.5.0.6. Table 6 compares the amplitude of the vibrations collected by each accelerometer. LSM9DS1 and ref. [40] have the same amplitude of vibrations.

The vibration frequencies from different road conditions are provided in Table 7. The dominant vibration frequency is approximately 5 Hz, which masks the vibration frequency of the vehicle itself because of the higher amplitude of vibrations in driving mode. In the highway and the bumpy road, there are other frequencies, but the resonances and vibrations are not as strong in those frequencies as the dominant 5 Hz frequency. There are different oscillations on the highway and bumpy road; however, the strongest oscillation is approximately 5 Hz, which makes it the main resonance obtained from the vehicle dashboard in driving mode. The test results for ADXL335 in [12] indicate that by placing the sensor in different locations, including the engine and dashboard, the amplitude of vibrations is 1.22 and 0.14 g, which is consistent with the 1.5 and 0.07 g amplitudes in this paper. The difference between the two is a result of using different makes and models of vehicles.

**Figure 18:**
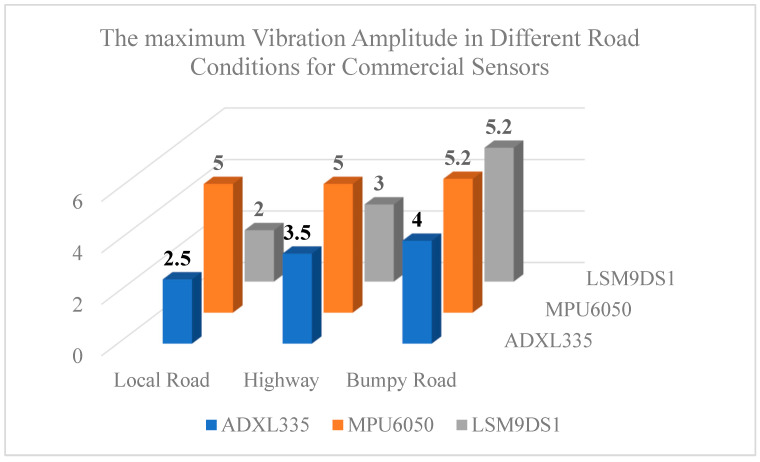
Comparison of the maximum vibration amplitude sensed by the commercial sensors under different road conditions.

Figure 18 shows the maximum vibration amplitude sensed by the commercial sensors under different road conditions. As seen here, the vibration amplitudes are the highest on bumpy roads. There is a slight increase in highway vibrations compared to local road vibrations. In total, the pattern of stronger vibrations can be seen in all the sensors, which is related to the condition of potholes and the bumpiness of the road.

## 5. Conclusions

This paper conducts a detailed experimental analysis and viability review of three commercial accelerometers and a customized accelerometer sensor to study their applicability in capturing the vibration dynamics in various locations on a vehicle under different idle and driving conditions. Moreover, this extensive study provides information that helps researchers customize new sensors by identifying the frequencies that should be selected for their design to separate the frequencies of both vehicle and road vibrations from that of the sensor. The results presented in this paper show the importance of inertial sensors in automotive dynamic sensing that are currently under-researched. A lab-designed in-house MEMS accelerometer and three commercial accelerometers are used to test the vehicle dynamics. Then, the output time domain frequency domain and PSD data are presented. The plots that display the output results provide an accurate representation of the dynamic conditions observed in the experiment. The frequency of the vibrations for the hood above the engine is 44.18 Hz for the in-house sensor and 40 Hz for the commercial sensors. The main vibration frequency of the hood above the radiator fan is 38 Hz, which is expected for the tested vehicle make and model. Conversely, the vibration frequencies of the road tend to fall within the lower frequency range of approximately 5 Hz. As expected, the low-frequency vibrations are dominant in driving mode and mask the motions from the vehicle itself since they are stronger and have higher amplitude. There is a slight difference in the frequency of the local road and bumpy road, being 5.03 Hz and 4.88 Hz, respectively. When conducting the test on the bumpy road, the lower speed of the car compensated for the expected increase in frequency. This is because, in such tests, the speed and frequency are typically proportional. The acceleration amplitude in the idle mode test ranges from 0.07 g to 2.5 g. The amplitude information is especially crucial when selecting a sensor. Trying to measure a vibration signal outside the sensor range can clip or distort the sensor output signal. The tested accelerometers have different sensitivities and response amplitudes. This study provides extensive information and comparison aiding in understanding how to select a specific sensor. The sensors used here are selected because of their compatibility with the test types in this paper and are not promoted. The higher sensitivity can be useful, especially in digitizing the response since there will be less possibility of error. Moreover, the detection of the signal will be simple. During the collection of dynamic data in driving mode, the time domain and FFT data show the driving conditions of the traffic and road surface, which proves that the sensitivity of the sensors is adequate to detect such conditions. These tests (altogether) are useful in condition monitoring for fault detection and system health investigations where vehicle health is concerned. The frequencies of the three road conditions are approximately 5 Hz, with different amplitudes for different levels of bumpiness. In higher amplitude vibrations, low-sensitivity accelerometers would be used. Both idle and drive mode test data provide valuable information that can be used for various purposes. These purposes include but are not limited to functionality, efficiency, comfortability of new smart cars, diagnostic, maintenance, and safety of the vehicles, which is feasible by gaining accurate and precise data on vehicles.

## Figures and Tables

**Table 1:** Accelerometer Sensor Specifications.

Accelerometer Specifications	MEMS ADXL335EB Sensor	MEMS MPU6050 Sensor	MEMS LSM9DS1 Sensor	MEMS in House Sensor	Piezoelectric Reference Standard 8305
Measurement Range	±3 g	±8 g	±8 g	±8 g	±100 g
Power Consumption	150 μA (typical)	3.9 mA (max.)	4.5 mA (typical)	5 mA	NA
Specified Voltage	3 V	+3 V to +5 V	1.9 V to 3.6 V	5 V	NA
Temperature Range	−40 to 85 °C	−40 to 85 °C	−40 to +85 °C	−40 to +85 °C	−74 to +200 °C

**Table 2:** Arduino UNO technical data information.

Manufacturer	Arduino
Microcontroller	ATMega328p
Operating voltage	5 V
Input voltage limit (recommended)	6–20 V (7–12 V)
Digital I/O pins	14 (of which 6 provide PWM output)
PWM digital I/O and Analog input pins	6
DC current per I/O pin	20 mA
DC current for 3.3v pin	50 mA
Flash memory	32 KB (0.5 KB used by bootloader)
SRAM	2 KB (ATmega328P)
EEPROM	1 KB (ATmega328P)
Clock speed and LED Built-in	16 MHz and 13

**Table 3:** Raspberry Pi technical data information.

Manufacturer	Raspberry Pi
Place of Business	CHICAGO, IL, 60693 US
Model and Part number	SC15184
Memory Storage Capacity and RAM	2 GB
Memory Slots Available	4
Memory Technology	SDRAM
Maximum Memory Supported	2 GB
RAM Technology	LPDDR4, SDRAM
Memory Type	DDR3 SDRAM
Processor Type and Number of Processors	Cortex, 4
OS and Hardware Interface	Linux, USB, USB Type C, Ethernet, HDMI, Video, USB 3.0, USB 2.0

**Table 4:** The detailed test plan for data collection of a vehicle in several dynamic conditions.

Test Number	The Application of Accelerometer Data	Laboratory Sensor and IMU Mounting Position	Driving Mode/Condition	Time (s)
1	To obtain the response from the engine	On the hood above the engine	Engine started, Idle mode	120
2	To obtain the response from the radiator fan	On the hood above the radiator fan	Engine started, Idle mode	120
3	To obtain the response from exhaust pipe	On trunk over the exhaust exit	Engine started, Idle mode	120
4	To obtain the response from dashboard	On the car dashboard	Engine started, Idle mode	120
5	To obtain the response from frequent braking	On the car dashboard	Driving mode on local roads	120
6	To measure acceleration in rapid speed changes	On the car dashboard	Driving mode on the highway	120
7	To observe the road condition	On the car dashboard	Driving mode on a bumpy country road	120

**Table 5:** The vibration frequencies in different locations on the vehicle in idle mode in Hz.

	Location on the Car	Hood above the Engine	Hood above the Fan	Exhaust Pipe	Dashboard
Sensor Type					
Lab-designed		44.18	23 and 38	10 and 27	22.8
ADXL335		40	38 and 75	22 and 28	24
MPU6050		40	38 and 75	22 and 28	24
LSM9DS1		40	38 and 75	22.6 and 28.6	24

**Table 6:** The amplitudes of vibrations on the dashboard in g.

	Location on the Car	Dashboard
Sensor Type		
Lab-designed		0.07
ADXL335		0.07
MPU6050		0.1
LSM9DS1		0.15
Ref. [40]		0.15

**Table 7:** The vibration frequencies of commercial sensors under different road conditions in Hz.

	Road Cond.	Local	Highway	Bumpy
Sensor Type				
ADXL335		5.03	5 and 66	4.88 and 18
MPU6050		5.03	5 and 64	4.88 and 17
LSM9DS1		5.03	5 and 60	4.88 and 17

## Data Availability

The data relating to this paper are available on request from the corresponding author.
